# Effect of prenatal antibiotics on breast milk and neonatal IgA and microbiome: a case-control translational study protocol

**DOI:** 10.1038/s41390-025-03922-4

**Published:** 2025-02-18

**Authors:** Carlo Pietrasanta, Andrea Ronchi, Carolina Carlosama, Michela Lizier, Alessandra Silvestri, Giulia Fornasa, Alessia Melacarne, Martin Mihula, Francesco D’Ambrosi, Martina Lutterotti, Elisa Carbone, Irene Cetin, Monica Fumagalli, Enrico Ferrazzi, Giuseppe Penna, Fabio Mosca, Lorenza Pugni, Maria Rescigno

**Affiliations:** 1 Department of Clinical Sciences and Community Health, Department of Excellence 2023-2027, University of Milan, Milan, Italy; 2 NICU Fondazione IRCCS Ca’ Granda Ospedale Maggiore Policlinico, Milan, Italy; 3 IRCCS Humanitas Research Hospital, Milan, Italy; 4 Department of Medical Biotechnology, Università di Siena, Siena, Italy; 5 Department of Woman, Child and Neonate, Fondazione IRCCS Ca’ Granda Ospedale Maggiore Policlinico, Milan, Italy; 6 University of Milan, Milan, Italy; 7 Department of Biomedical and Clinical Sciences, University of Milan, Milan, Italy; 8 Department of Biomedical Sciences, Humanitas University, Milan, Italy

## Abstract

**Background:**

Up to 25–35% of women receive antibiotics (ABX) during pregnancy, but little is known about the consequences on a key mucosal interface such as the mammary gland, and on the development of the neonatal gut’s microbiota and IgA. We hypothesize that prenatal ABX negatively affect the immune functionality of mammary gland, the composition of breast milk microbiota, the development of neonatal fecal microbiota and the abundance of neonatal fecal IgA.

**Methods:**

Case-control translational cohort study on women and neonates in the presence or absence (*N* = 41 + 41 pairs) of exposure to prenatal ABX for at least 7 consecutive days after 32 weeks of gestation.

**Results:**

We will evaluate IgA concentration in breast milk and in neonatal feces up to one year after delivery. We will also evaluate clinical parameters, neurodevelopment and the composition of the IgA-coated and uncoated fractions of breast milk and fecal microbiota by means of magnetic-activated cell sorting (MACS) coupled with shotgun metagenomics. Finally, we will measure the concentration of the chemokine CCL28 on maternal serum and breast milk, as a marker of activity of the entero-mammary pathway.

**Conclusions:**

Our results might support a data-driven evaluation of breast milk immune function in women exposed to prenatal ABX.

**Impact:**

- Breast milk IgA and microbiota are critical to determine the positive effects of breastfeeding in infants.
- This research protocol will investigate breast milk IgA, microbiota, and the IgA^+^ / IgA^−^ fractions of neonatal fecal microbiota upon exposure to prenatal antibiotics.
- Fecal IgA and microbiota in infants exposed or not exposed to prenatal antibiotics will be analyzed up to 1 year after birth.
- This research will clarify the impact of prenatal antibiotics on the immune function of breast milk.
- This, in turn, might support the selective evaluation of breast milk IgA/microbiota in mothers exposed to prenatal antibiotics, or in donor human milk.

## Introduction

At the mucosal sites of human body, the relationship between the microbiota and the immune system is mutual: the abundance and diversity of microbiome can affect the function of immune system, while the components of the mucosal immune system, such as secretory IgAs (sIgA), can in turn shape the microbiota composition.^1–5^ Indeed, it is known that humans deficient in IgA have a unique intestinal microbiota characterized by the expanded of *Enterobacteriaceae*,^6^ akin to IgA-deficient murine strains where *Enterobacteriaceae and* segmented filamentous bacteria (SFB) are predominant.^7,8^ In early life, breast milk microbiota and breast milk immune components, such as lactoferrin and sIgA excreted by the mammary gland plasma cells, play key roles in shaping the assembly of neonatal intestinal microbiota and contribute to the passive protection of neonates against bacterial translocation from the gut.^3,9–11^ The positive effects of breast milk on infants’ health might not be limited to the gut health or to the breastfeeding period: a growing body of evidence reveals that breastfeeding probably has a positive influence on neurodevelopmental long-term outcomes compared to formula feeding in both term-^12,13^ and preterm-born children,^14^ and on the body composition and growth of children even beyond the breastfeeding window.^15,16^ These effects might be largely contributed by the unique intestinal microbiota induced by breastfeeding, and by the immune components of breast milk themselves.^17,18^

Antibiotic (ABX)-induced dysbiosis is known to disrupt the immune function of different mucosal sites, such as the gut and the lungs of adult individuals,^19^ but the effect on the mammary gland immune system is not known. Moreover, ABX administered directly to neonates during the first days of life are also known to induce neonatal dysbiosis and are associated with short- and long-term immune-related adverse outcomes such as an increased incidence of necrotizing enterocolitis (NEC), atopy, and obesity.^20–22^ Despite such an extensive knowledge on the effects of postnatal ABX, the effect of prenatal ABX-induced dysbiosis on the breast milk immune function and on the development of mucosal neonatal immune system is poorly understood. Our recently published data on a murine model of prenatal ABX exposure showed that maternally administered ABX during the last week of pregnancy decrease both maternal and neonatal mucosal IgA and induces a long-term derangement of neonatal fecal microbiota.^23^ A recent, not peer-reviewed work confirmed our findings.^24^ We hypothesize that prolonged ABX therapy (≥7 days of therapy) administered to pregnant mothers during the last stage of pregnancy, i.e., shortly before birth, can reduce the absolute amount and the polyreactivity of breast milk IgA after birth, even without any administration of ABX during the breastfeeding period. This reduction could be caused by an impaired functionality of the so-called “entero-mammary pathway”, through which plasma cells migrate to the mammary gland from the mesenteric lymph nodes driven by the gradient of the epithelial chemokine CCL28 produced by the mammary gland itself.^25^ Therefore, we hypothesize that maternal intestinal dysbiosis during the last stages of pregnancy can impair the migration of IgA-producing plasma cells to the mammary gland. Moreover, most prescribed ABX in obstetric clinical practice, such as penicillins and macrolides, are easily absorbed and can diffuse in all body tissues. We thus hypothesize that breast milk microbiota itself might be profoundly altered by prenatal ABX.

Finally, during the breastfeeding window plasma cells of the neonatal intestinal mucosa produce limited amounts of IgA, and the concentration of fecal IgA in neonates depends almost exclusively on the passive transmission of maternal IgA through breast milk.^26^ Similarly, the composition of neonatal intestinal microbiota is greatly dependent on the type of milk received (breast milk or formula milk). We thus also hypothesize that the reduction in breast milk IgA and the alteration of breast milk microbiota induced by prenatal ABX can cause a reduction in neonatal fecal IgA, and a condition of neonatal intestinal dysbiosis favored by microbially-altered maternal breast milk.

## Methods/design

### Study design

**Fig. 1:**
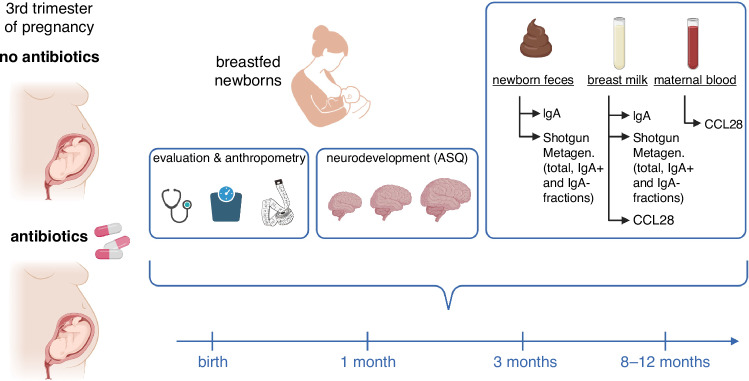
Graphical overview of the study protocol and procedures. ASQ: Ages and Stages Questionnaires. CCL28: Chemokine (C-C motif) ligand 28.

The overall study design is summarized in Fig. 1. The study will be performed at the Department of Woman, Child and Newborn of Fondazione IRCCS Ca’ Granda—Ospedale Maggiore Policlinico of Milan, Italy, with the experimental collaboration of the Mucosal Immunology and Microbiota laboratory of IRCCS Istituto Clinico Humanitas, Rozzano (Milan), Italy. The protocol has been registered at ClinicalTrials.gov, trial code: NCT05813184. Registered on April 14, 2023. This will be a prospective case-control study enrolling pregnant women between 18 and 40 years of age exposed to antibiotic therapy during the last month of pregnancy, for a period of at least 7 days consecutively, and their newborns.

Conditions for which women will be treated with prolonged antibiotics will include but will not be limited to prelabor or preterm-prelabor rupture of membranes (PROM or pPROM), preterm labor with or without PROM, urinary tract infections, clinical chorioamnionitis, pneumonia. An equal number of women and their neonates not exposed to prenatal antibiotics (any exposure during pregnancy) will be enrolled as controls. Case-control dyads will be matched for gestational age at delivery and at the start of antimicrobial treatment.

Inclusion criteria will be the expression of written informed consent by parents of the neonate, a maternal antibiotic treatment (any molecule) for at least 7 days consecutively after 32 weeks of gestational age (or the absence of exposure to any systemic antibiotic treatment during pregnancy for the control group), and the intention to breastfeed neonates as long as possible during the first year of life. Exclusion criteria will be the absence of written informed consent, the intention or medical need to formula feed exclusively (or the presence of significant maternal concerns about breastfeeding), a maternal antibiotic treatment shorter than 7 days, the presence of pre-existing maternal immune-mediated disorders (including chronic immunodeficiencies), a delivery at a gestational age <34 weeks and the administration of antibiotics to neonates after birth, within the first week of life.

The amount of IgA (in breast milk and neonatal feces), the composition of microbiota (in breast milk and neonatal feces), the proportion and profile of IgA-coated bacteria (in neonatal feces), and the concentration of the Chemokine (C-C motif) ligand 28 (CCL28, in maternal serum and breast milk) will be quantified after collection of samples during pre-planned study visits, at the following time points:

1. during the first week of life
2. at 1 month of life
3. at 3 months of life

at 8–12 months of life (or at the time of breastfeeding interruption), after the introduction of solid food in the infant’s diet. At the same timepoint, five domains (communication, fine motor, gross motor, problem-solving ability, and personal-social functioning) of infants’ neurodevelopment will be assessed through the administration of Ages and Stages Questionnaire-3 (ASQ-3), and infants’ growth will be evaluated (weight, length, head circumference). The research protocol was reviewed and approved by Comitato Etico Milano Area 2, Approval number 0007973. Written informed consent will be obtained from enrolled women and the fathers of enrolled neonates. The study protocol adheres to the STROBE checklist for case-control studies and was registered at ClinicalTrials.gov (protocol code: NCT05813184, registered on April 14, 2023).

### Laboratory methodologies

Breast milk will be manually expressed by trained nurses or by the mothers themselves after cleansing of the breast skin. Feces will be collected from the diaper using a sterile plastic spatula. All biological samples will be collected in sterile containers and immediately frozen at −80 °C (samples collected during hospital stay and study visits), or at −20 °C for samples collected at home (neonatal feces during follow up) and subsequently transferred at −80 °C. This will allow to minimize alterations of IgA pool and microbiota composition. For experimental analysis samples will be defrosted in batches, always with an equal number of samples between experimental groups.

IgA quantification in breast milk will be performed with commercially available ELISA kit (Invitrogen, cat. BMS2096), in technical duplicates and including a standard curve in each 96-well plate. Breast milk samples will be devoid of fat components by centrifugation and appropriately diluted in assay diluent. Feces will be defrosted, suspended in PBS with 0.1 mg/ml of trypsin inhibitor from soybean (Sigma Aldrich) at a concentration of 100 mg/ml, and homogenized through shaking at 30 Hz for 20 s in a tissue lyser (Qiagen), after the addition of 1.4 mM ceramic beads (MP Biomedicals). Then they will be centrifuged at 50 × *g* for 10 min to remove debris, and supernatant will be diluted 1:5 with assay diluent.

For magnetic-activated cell sorting (MACS) of IgA-coated and non-coated bacteria, defrosted, homogenized, and diluted pellets will be slowly centrifuged (50 × *g* for 10 min, 4 °C) to remove large particles, leaving bacteria in suspension. Supernatants will be collected and spun down at 4000 × *g* × 10 min (4 °C) to retrieve bacterial pellets, that will be then resuspended in MACS buffer, blocked with anti- CD16/32 solution in MACS buffer (20 min, 4 °C) and stained with anti-human IgA PE-conjugated antibody (clone mA-6E1, 1:100) for 30 min at 4 °C. After appropriate washing steps, stained pellets will be incubated in 1 ml staining buffer containing 50 μl Anti-PE Magnetic Activated Cell Sorting (MACS) beads (Miltenyi Biotec). This solution will then be sorted by MACS through LS columns (Miltenyi) to separate IgA^+^ and IgA^-^ fractions of fecal microbiota. For sequencing of bacteria from feces and milk, DNA will be extracted withDNeasy PowerSoil Pro Kit (Qiagen, cat. 47019) and sequenced by shotgun metagenomics on the Illumina HiSeq 2500 platform by Prebiomics S.r.l., Trento, Italy. The quality of raw sequencing data will be assessed using MultiQC (v1.11).^27^ Host contaminant reads (human DNA) will be filtered out using KneadData (v0.10.0) with Bowtie2 (v2.5.4)^28^ against the GRCh38 genome reference database. Taxonomic profiling will be performed using MetaPhlAn (v4.0.6), and functional profiling will be conducted using HUMAnN (v3.9). For downstream analysis, the QIIME2 platform (v2024.10) will be employed.^29,30^ Alpha and beta diversity metrics will be calculated with the qiime diversity core-metrics-phylogenetic command. Shannon Entropy will be used as representative metrics for alpha diversity, with Box and Whisker plots generated in GraphPad Prism (v10.2.3). BrayCurtis dissimilarity will be used as a representative metric for beta diversity, and a Principal Coordinate Analysis (PCoA) plot will be generated in R (v4.3.1) using the “ggplot2” library.

Chemokine CCL28 will be measured on maternal serum and breast milk with commercially available ELISA kit (Bio-Techne, cod. DY717). Appropriate negative and positive controls will be carried on throughout all the protocol steps.

### Data collection, sample size and statistical analysis

Demographic and clinical data will be collected prospectively in an electronic database (Excel software, Microsoft Corp.), where enrolled patients will be de-identified and associated with a single numerical code. The database will be stored on the Institutional server and encrypted according to standard internal protocols for data protection.

According to internal historical data, an average of 1.5 women per week is expected to receive prolonged ABX therapy immediately before delivery at the study site. Based on preliminary animal data, an average drop of approximately 30% in breast milk IgA can be expected after prenatal ABX treatment. Considered an average content of IgA of 3.8 g/L (S.D. ± 1 g/L), a sample size of around 34 women per group would be enough to detect a prudential difference of 20% with a power of 0.9 and an Alpha equal to 0.05. We would like to account for a dropout rate of 20%. Therefore, a sample size of 41 women per group (ABX-treated vs. controls) would be appropriate and feasible within one year in our clinical context.

Results will be represented using box plots showing the interquartile range, with a single data point per subject. Statistically significant differences between two groups will be evaluated with Mann-Whitney two-sided unpaired U-test, for non-normally distributed data (visually checked), or two-sided unpaired *t*-test for normally distributed data. In case of longitudinal experiments (e.g., IgA concentration over time in the same subjects), repeated-measures ANOVA will be applied. A probability value of *p < 0.05 will be considered as significant. Data analysis and graphing will be performed using GraphPad Prism (GraphPad software) vers. 9.0.1, and StataMP vers. 17.0 (Stata Corp.).

### Timing of analysis

The enrollment will last 12 months, as well as follow up for each mother-infant dyad enrolled. Samples and data analysis will also last 12 months in total, subdivided in 4 periods of 3 months (every 6 months) to ensure ad-interim evaluation of results.

## Discussion and conclusions

Early life is a key period for the development of future health.^31^ As previously shown by others, perinatal perturbations to the physiological interaction between the new assembling microbiota and the developing neonatal immune system can have devastating short- and long-term consequences.^10,32,33^ The administration of ABX to neonates is associated with an increased risk for death, NEC, antibiotic resistance, and later obesity.^20,34,35^ However, ABX are administered to a minority of neonates, especially those born prematurely. Conversely, at least 1 every 4 women receive ABX during pregnancy, but the effects of these therapies on breast milk immune function and on the development of neonatal mucosal immune system, in particular on IgA-producing plasma cells of the mammary gland (in treated women) and of the intestinal mucosa (in breastfed neonates), remain largely unknown. Published research on the effects of prenatal antibiotics on newborn health has been mainly focused on the vertical transmission of microbiota from the mother to her offspring, and how alterations of this process affect neonatal outcomes. Little is known about negative consequences of prenatal antibiotics on the pool of maternal antibodies, mainly IgA, transferred to the infant, and on how these together with an altered inherited microbiota shape the maturation and function of neonatal mucosal IgA-producing plasma cells themselves. On a murine model, we have recently demonstrated that pups born to antibiotic-treated mothers have reduced fecal SIgA and IgA-coated bacteria, making them more susceptible to bacterial translocation from the gut and that a “low breast milk IgA phenotype” has long-lasting negative effect on IgA-producing plasma cells of the neonatal gut.^23^ Thus, maternal breast milk IgA and microbiota can shape the later function of small intestine IgA-producing plasma cells themselves. In humans, Gopalakrishna et al. have shown that maternally-derived IgA is key for the protection of preterm neonates against NEC, coating bacterial species of the Enterobacteriaceae family that are associated with the occurrence of NEC.^36^ Finally, a preliminary report (not peer-reviewed yet) on two cohorts of women who delivered prematurely and their neonates shows lower concentrations of breast milk IgA in a small sample of women who received antibiotics before delivery.^24^ None of these cohorts was followed up longitudinally throughout the first year of life.

Our study has been planned as a case-control, proof-of-concept mechanistic study on late-preterm and term-born neonates and their mothers. The case-control study design has been chosen for two reasons: first, to better control and balance the enrolled populations (exposed or not to prenatal antibiotics) for clinically relevant variables such as gestational age and breastfeeding rates, and second to limit the size of the enrolled cohorts and thus the costs of cutting-edge laboratory analysis we will perform on different biological samples at multiple timepoints. Moreover, we decided to focus our research on late-preterm and term-born neonates, even though several neonates exposed to prenatal antibiotics are born severely or extremely preterm. Indeed, the majority of these small neonates are also exposed to postnatal antibiotics, and this makes it cumbersome to ascertain the biological consequences of prenatally administered antibiotics. A limitation of our study is that it will not be adequately powered to analyze the clinical outcomes of infants exposed or not exposed to prenatal Abx. In the future, larger cohort studies might be able to achieve this goal, focusing on the clinical consequences of prenatal Abx on outcomes such as the rates of gastrointestinal infections, food allergies or intestinal inflammatory diseases.

Considered the tight interplay between dysbiosis and the functionality of immune system repeatedly shown in other contexts,^37,38^ to identify adverse effects of prenatal antibiotics on neonates and possible beneficial interventions might be relevant for the health of neonates born to ABX-treated mothers. Our project will shed new light on these aspects, and the results will hopefully represent a keystone for a better comprehension of a previously underestimated phenomenon: the adverse effects on breast milk and neonatal gut of antibiotics administered to mothers before birth.

## Data Availability

All data generated or analyzed during this study are summarized in this article. Further enquiries can be directed to the corresponding author.
