# Correction: Effect of prenatal antibiotics on breast milk and neonatal IgA and microbiome: a case-control translational study protocol

**DOI:** 10.1038/s41390-025-04318-0

**Published:** 2025-08-05

**Authors:** Carlo Pietrasanta, Andrea Ronchi, Carolina Carlosama, Michela Lizier, Alessandra Silvestri, Giulia Fornasa, Alessia Melacarne, Martin Mihula, Francesco D’Ambrosi, Martina Lutterotti, Elisa Carbone, Irene Cetin, Monica Fumagalli, Enrico Ferrazzi, Giuseppe Penna, Fabio Mosca, Lorenza Pugni, Maria Rescigno

**Affiliations:** 1 Department of Clinical Sciences and Community Health, Department of Excellence 2023–2027, University of Milan, Milan, Italy; 2 NICU Fondazione IRCCS Ca’ Granda Ospedale Maggiore Policlinico, Milan, Italy; 3 IRCCS Humanitas Research Hospital, Milan, Italy; 4 Department of Medical Biotechnology, Università di Siena, Siena, Italy; 5 Department of Woman, Child and Neonate, Fondazione IRCCS Ca’ Granda Ospedale Maggiore Policlinico, Milan, Italy; 6 University of Milan, Milan, Italy; 7 Department of Biomedical and Clinical Sciences, University of Milan, Milan, Italy; 8 Department of Biomedical Sciences, Humanitas University, Milan, Italy

Correction to: Pediatric Research 10.1038/s41390-025-03922-4, published online 18 February 2025

In this article, Martin Mihula at affiliation ‘IRCCS Humanitas Research Hospital, Milan, Italy’ and ‘Department of Medical Biotechnology, Università di Siena, Siena, Italy’ was missing from the author list.

Affiliations have been rearranged to reflect the corrected author list.

In the sentence beginning ‘The quality of raw sequencing data......’ in this article, the text should have read ‘The quality of raw sequencing data will be assessed using MultiQC (v1.11).^27^ Host contaminant reads (human DNA) will be filtered out using KneadData (v0.10.0) with Bowtie2 (v2.5.4)^28^ against the GRCh38 genome reference database. Taxonomic profiling will be performed using MetaPhlAn (v4.0.6), and functional profiling will be conducted using HUMAnN (v3.9). For downstream analysis, the QIIME2 platform (v2024.10) will be employed.^29,30^ Alpha and beta diversity metrics will be calculated with the qiime diversity core-metrics-phylogenetic command. Shannon Entropy will be used as representative metrics for alpha diversity, with Box and Whisker plots generated in GraphPad Prism (v10.2.3). BrayCurtis dissimilarity will be used as a representative metric for beta diversity, and a Principal Coordinate Analysis (PCoA) plot will be generated in R (v4.3.1) using the “ggplot2” library’.

The original article has been corrected.

